# Supplementary material for: Predictors of Response to a Medial Branch Block: MRI Analysis of the Lumbar Spine
Source: J Clin Med. 2019 Apr 19;8(4):538. doi: 10.3390/jcm8040538 (PMC6518052; doi:10.3390/jcm8040538)
Supplement: Supplementary file 1 [file jcm-08-00538-s001.pdf]

Supplement Table S1. Spearman’s rank correlation coefficients and their statistical significance between the numeric rating scale and facet angle difference at L3–4, disc height at L5–S1.

Variables	L3-4 facet angle difference		L5-S1 disc height	
	Spearman correlation coefficient	P value	Spearman correlation coefficient	P value
NRS difference (points)	0.121	0.168	-0.209	0.017
NRS change (%)	0.163	0.062	-0.206	0.019

NRS difference is defined by subtracting one-month NRS after MBB from baseline NRS. NRS change is defined by the NRS difference divided by baseline NRS. NRS = numeric rating scale; MBB = medial branch block
